# Assessment of glomerular filtration rate in trauma patients in early resuscitation phase

**DOI:** 10.1186/cc10964

**Published:** 2012-03-20

**Authors:** M Bhattacharyya, R Kumar, S Todi

**Affiliations:** 1 AMRI Hospitals, Kolkata, India; 2 Jadavpur University, Kolkata, India

## Introduction

An estimate of the glomerular filtration rate (GFR) is important to individualize drug dosages. Trauma leads to systemic inflammatory response syndrome, which has an effect on GFR. The main objective of this study was to assess the GFR in trauma patients during the first 24 hours of admission.

## Methods

A prospective observational study of 50 trauma patients aged between 18 and 90 years admitted to the ICU. Exclusion: patients with chronic kidney disease and structural kidney damage. The study population was assessed for GFR by the measurement of creatinine clearance from 24-hour urine creatinine and from serum creatinine. Demographic parameters were documented.

## Results

Total patients admitted to the ITU during July 2010 to April 2011 with trauma were 67, of which 50 patients were included in the study. The mean age of the study group was 39 years, male 86%, mean APACHE IV score 32 and mean Injury Severity Score 10. Out of 50 trauma patients, 13 (26%) patients developed glomerular hyperfiltration (GHF) within 24 hours of admission. Mean creatinine clearance in the GHF group was 177.92 ± 29 and minimum/maximum values were 151.4 and 254.3 ml/minute/1.73 m^2 ^respectively. Compared to the GHF group, mean creatinine clearance levels were considerably lower in non-GHF patients (86.03 ± 29) with a range of values from 41 to 138.5 ml/minute/1.73 m^2^.

## Conclusion

Incidence of glomerular hyperfiltration is relatively common in critically ill multitrauma patients in the first 24 hours. This should be taken into account while deciding drug dosing in this group of patients.

